# Rapid Freeze-Quench EPR Spectroscopy: Improved Collection of Frozen Particles

**DOI:** 10.1007/s00723-016-0783-7

**Published:** 2016-04-30

**Authors:** Faezeh Nami, Peter Gast, Edgar J. J. Groenen

**Affiliations:** Huygens-Kamerlingh Onnes Laboratory, Department of Physics, Leiden University, P.O. Box 9504, 2300 RA Leiden, The Netherlands

## Abstract

Rapid freeze-quench (RFQ) in combination with electron paramagnetic resonance (EPR) spectroscopy at X-band is a proven technique to trap and characterize paramagnetic intermediates of biochemical reactions. Preparation of suitable samples is still cumbersome, despite many attempts to remedy this problem, and limits the wide applicability of RFQ EPR. We present a method, which improves the collection of freeze-quench particles from isopentane and their packing in an EPR tube. The method is based on sucking the particle suspension into an EPR tube with a filter at the bottom. This procedure results in a significant reduction of the required volume of reactants, which allows the economical use of valuable reactants such as proteins. The approach also enables the successful collection of smaller frozen particles, which are generated at higher flow rates. The method provides for a reproducible, efficient and fast collection of the freeze-quench particles and can be easily adapted to RFQ EPR at higher microwave frequencies than X-band.

## Introduction

Detection and characterization of intermediates involved in enzymatic reactions is an important step to understand the mechanism of such reactions. Elucidating the reaction kinetics and the structure of enzymatic intermediates commonly requires a combination of spectroscopic techniques in view of the complexity of the systems [1]. Except for the optical stopped-flow technique, spectroscopic characterization necessitates to trap intermediates before observation. Rapid freeze-quench (RFQ) [2, 3] is a proven technique to trap the intermediates on the time scale of milliseconds. In RFQ the reaction components are rapidly mixed and after a certain time the reaction is instantly quenched in a cryomedium. The RFQ technique has been mostly combined with electron paramagnetic resonance (EPR) spectroscopy, because enzymatic reactions often involve a radical or a transition metal ion [4–6]. An EPR spectrum can provide insights into the nature of the paramagnetic center, its conversion during the reaction and its interaction with the chemical environment. The mechanism of a large number of enzymatic reactions, in particular copper- and iron-containing enzymes, has been studied by RFQ EPR techniques [7–14].

Over the last two decades, the RFQ technique has also been combined with other spectroscopic techniques to complement RFQ EPR spectroscopy or to study intermediates that do not show an EPR signal. Mössbauer spectroscopy in combination with RFQ has been used for kinetic and mechanistic studies of iron proteins such as *E. coli* ribonucleotide reductase [15], taurine:α-ketoglutarate dioxygenase [16, 17], methane monooxygenase [18, 19], and cytochrome p450 [20]. X-ray absorption spectroscopy in combination with RFQ has been used to examine the structure of a number of metalloprotein reaction intermediates [19, 21] including zinc-containing intermediates, which are silent in EPR [22]. Developments in sample preparation allowed combination of the RFQ technique with a wider variety of spectroscopic techniques such as resonance Raman [23] and magnetic circular dichroism [24].

Rapid freeze-quench dates back to 1961, when Bray [2] introduced a technique called “thermal quenching” for the study of the kinetics of fast reactions by means of EPR spectroscopy. The traditional RFQ apparatus consists of three components: the ram unit, the syringes and the reaction unit. The reagents are pushed out of the syringes into the mixing chamber, and subsequently the reaction mixture is sprayed into isopentane at −140 °C. In the first experiments, the dead time of the RFQ setup was 30 ms and a sample volume of 300–500 µl was required for each time point [2]. Since then, a number of modifications has been made to try and improve the performance of RFQ instruments, particularly to reduce the amount of sample needed, the mixing time and the quenching time. The shortest quenching time of 50 µs was achieved using a micro-mixer in a home-built setup [25]. A serious difficulty, which has limited the application of the RFQ technique, is packing the small freeze-quench particles into an EPR tube. As a consequence of the inefficiency of the packing procedure, a large amount of sample is required, which might be prohibitive. Around 200 µl of solution mixture is typically sprayed into isopentane to end up about 1 cm of frozen particles in an EPR tube (i.d. ≥3 mm) [26]. Considering the packing density of approximately 0.5 [27–29], the collection of 200 µl of frozen particles should ideally result in a height of about 5 cm. This means that the efficiency of packing is about 20 % or, in other words, about 80 % of the frozen sample cannot be collected. In addition, irreproducible packing leads to a large variation in the magnitude of the EPR signal, which sensitively depends on the packing density and the amount of the sample in the tube. This irreproducibility of packing subsequently affects the accuracy of kinetic data obtained by the RFQ EPR technique [3, 27].

Many attempts have been made to remedy the packing problem. A low flow rate or a nozzle with a larger opening was used to produce larger frozen particles, which are easier to pack, but both resulted in less efficient quenching [30]. A pressure/filtration method was developed to force isopentane out of the particle suspension through a porous disk [28]. The particle suspension was concentrated in a cold centrifugation setup to obtain particles that are easier to collect and pack [31]. Different sample collectors were constructed to collect the frozen particles from isopentane [29]. Despite all efforts to improve the packing, collecting small freeze-quench particles from the cryogenic liquid remains challenging. The conventional way of packing using a packing rod is still mostly used, regardless of its low efficiency.

To overcome the packing limitation, we have developed an alternative method to collect the freeze-quench particles in an EPR tube. We suck the particle suspension into the EPR tube by pumping on the tube through a filter placed at the bottom of the tube. Isopentane exits through the filter, while the frozen particles are trapped in front of the filter. A more quantitative collection of the frozen particles results, which means a significant reduction of the amount of reactants required. The approach also makes the packing more reproducible, allows the use of a wide range of flow rates and reduces the packing time substantially. Last but not least, the method is easy and user-friendly.

## Materials and Methods

Myoglobin (Mb) from equine heart and sodium azide (NaN_3_) have been purchased from Sigma. Both Mb and NaN_3_ were dissolved in 100 mM sodium phosphate buffer, pH 7.8. The concentration of the myoglobin solutions was determined spectrophotometrically using an extinction coefficient *ɛ*_505_ = 9.7 mM^−1^ cm^−1^.

### Preparation of Rapid Freeze-Quench Samples

The rapid freeze-quench experiments have been performed using an Update Instrument System 1000 Chemical/Freeze Quench Apparatus with a Model 1019 syringe ram and a Model 715 ram controller. Two sizes of 0.5 and 2 ml glass syringe barrels were used. Before loading the reagents, the syringes were assembled and mounted on top of the syringe ram. Manual loading was used to have better control over the speed and, consequently, reduce the chance of drawing in air. Both syringes were filled simultaneously while the coupling hoses were inside their respective solutions during the entire loading process to prevent drawing in air. When the syringes were filled with the reagents, any air bubbles in the syringes and coupling hose were carefully expelled by moving the ram forward.

We used isopentane as cryogenic medium. Isopentane was first cooled down to the temperature of approximately −150 °C by pouring liquid nitrogen to it in a dewar. Pre-cooled nitrogen gas was used to keep the bath temperature at −135 to −140 °C. The temperature of the bath was monitored with a thermometer equipped with a Type K thermocouple (OMEGAETTE, model HH308). The isopentane bath was continuously stirred to ensure a homogeneous temperature. A glass tube (Duran, 12 ml) was filled with cooled isopentane and equilibrated in the isopentane bath. The opening of the tube was covered to minimize warming up. Once the isopentane in the glass tube reached the desired temperature, we started the sample preparation. The reagents were rapidly mixed and the reaction mixture was sprayed through the nozzle into the isopentane in the glass tube, which causes instantaneous quenching of the reaction.

### Sample Packing

The packing concerned sucking the particle suspension into an EPR tube with a filter at the bottom. Isopentane exits through the filter while the frozen particles remain behind in the tube. The EPR tubes (quartz, o.d. 4 mm, i.d. 3 mm, purchased from Heraeus Quarzglas GmbH) were cut to a length of 10 cm. Both sides of tubes were open and one side was tapered to keep the filter in the tube. The filter with a diameter of 2.8 mm was made by punching from a polypropylene disk purchased from Scientific Commodities. The filter had a thickness of 1.57 mm and a pore size of 35 μm and was pushed down to the bottom of the EPR tube using a steel rod. Once the filter was properly placed, the filter side of the EPR tube was connected to a water aspirator through latex tubing. The EPR tube assembly was precooled in cold isopentane at the temperature of dry ice. Then the glass tube containing the freeze-quench sample was quickly transferred from the isopentane bath to a Styrofoam box filled with dry ice. The open end of the EPR tube was inserted in the glass tube while the filter side was kept on dry ice to avoid warming up the sample. The particle suspension was sucked into the EPR tube by using the aspirator. Once the particles were inside the tube, they were more tightly packed using a steel rod. The aspirator was stopped when all liquid isopentane was sucked out of the tube. The EPR tube was disconnected from the aspirator by cutting the latex tube using a precooled cutter. The prepared samples were kept in liquid nitrogen until further use.

### EPR Measurements

Continuous-wave (CW) EPR spectra were obtained at 9.48 GHz using an ELEXSYS E680 spectrometer (Bruker BioSpin GmbH) equipped with a standard TE_102_ cavity and an ESR900 Cryostat (Oxford Instruments). The measurements were performed at 20 K, modulation amplitude/frequency of 0.5 mT/100 kHz, time constant of 40 ms and microwave power of 0.16 mW.

## Results

In order to investigate the effectiveness of the procedure to pack the rapid freeze-quench particles, we considered myoglobin and the binding reaction of myoglobin with azide, which is the commonly used test reaction for X-band RFQ EPR.

**Fig. 1:**
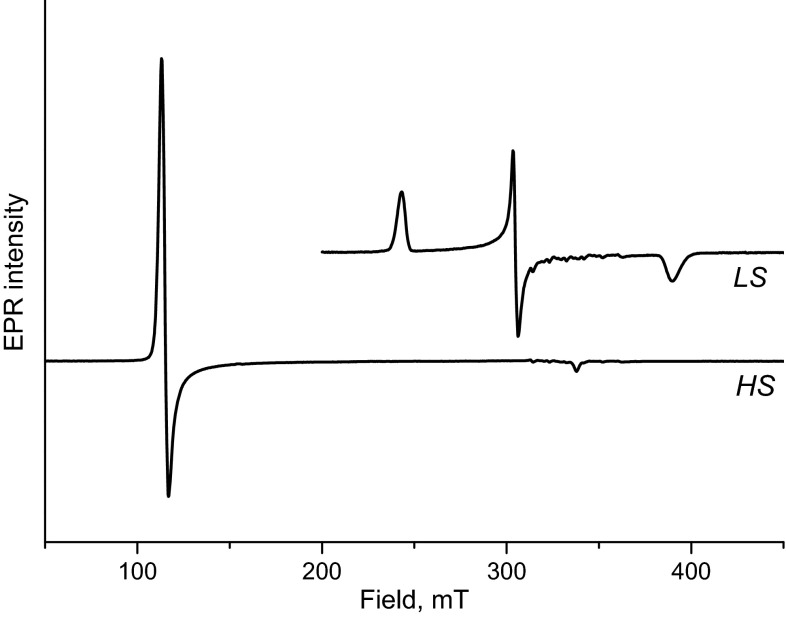
X-band EPR spectra of myoglobin in phosphate buffer, 100 mM, pH 7.8 (HS form), and of myglobin mixed with sodium azide, 30 mM (LS form). The small signals around 320 mT originate from Mn^2+^. EPR measurements: microwave frequency 9.48 GHz, temperature 20 K, microwave power 0.16 mW, modulation amplitude 0.5 mT and time constant of 40 ms

Myoglobin at neutral pH exists in the high-spin (HS) iron (Fe^3+^, *S* = 5/2) form. The HS form, in which one of the axial positions of iron is occupied by a water molecule, exhibits an EPR spectrum with a sharp signal at *g* = 6 and a small one at g = 2. The weakly bound water is readily replaced by exogenous ligands such as azide. This ligation change leads to the low-spin (LS) iron (Fe^3+^, *S* = 1/2) configuration, which gives rise to a different EPR spectrum with a rhombic g-tensor around *g* = 2. Figure 1 shows the X-band EPR spectra of the HS and LS forms of myoglobin. Below we report on the characterization of the packing procedure before we consider the test reaction.

### Evaluation of the Sucking Method

To evaluate the performance of the new packing method, the packing factor and collection efficiency are determined. The packing factor reflects the density of packing and the amount of isopentane included in RFQ prepared samples. The collection efficiency reflects the amount of freeze-quench material collected in the EPR tube relative to the amount of material sprayed into isopentane.

Six RFQ samples were prepared by mixing 800 µM myoglobin in 100 mM sodium phosphate buffer at pH 7.8 with the same buffer solution. The experiments were performed with the 2 ml syringes in 1:1 mixing mode and the flow rate of 2.7 ml s^−1^. For each sample, 150 µl of mixed solution was sprayed into isopentane. The collection of freeze-quench particles resulted in samples with a height of 2.6–2.9 cm in the EPR tube.

**Fig. 2:**
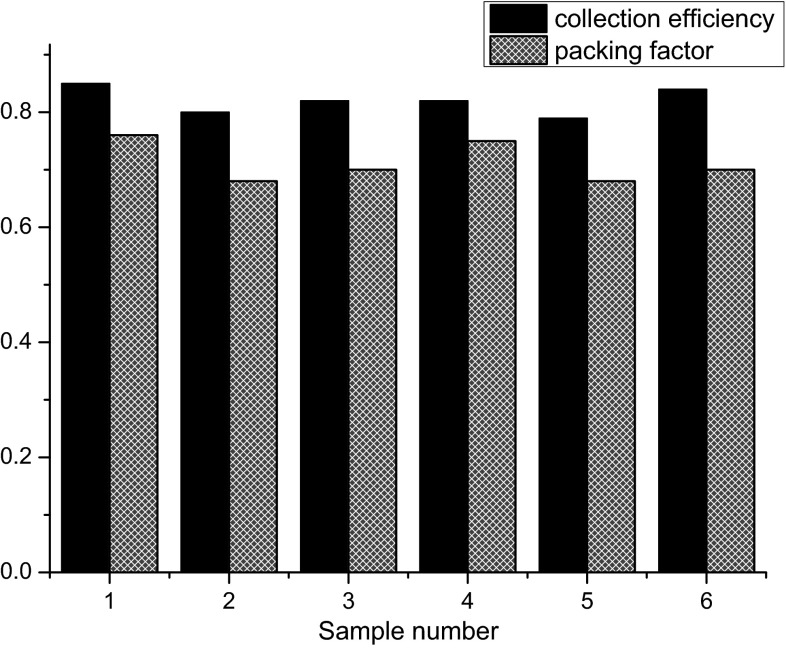
Collection efficiency and packing factor obtained for the sucking method. The rapid freeze-quench samples were prepared by mixing 800 µM myoglobin in 100 mM sodium phosphate buffer at pH 7.8 with the same buffer solution. For the experiments 2 ml syringes in 1:1 mixing mode and the flow rate of 2.7 ml s^−1^ were used

The packing factor is calculated from the ratio of the HS Fe^3+^ signal of the RFQ samples to that of the sample obtained by manually mixing of the same solutions. The packing factor varies between 0.68 and 0.76 (Fig. 2).

The collection efficiency is obtained from the ratio of the volume of a melted RFQ sample to that of the material sprayed into isopentane. The collection efficiency varies between 0.79 and 0.85 (Fig. 2).

We tried to pack freeze-quench particles prepared in the same way using the conventional packing method. It was found impossible to accumulate enough sample in the EPR tube with the conventional method for the small particles formed at the relatively high flow rate of 2.7 ml s^−1^.

In another set of experiments, we reduced the amount of mixed solution sprayed into isopentane to 60 µl. The experiments were performed using the 0.5 ml syringes in 1:1 mixing mode and the flow rate of 1.2 ml s^−1^ and we were able to fill 0.8–1 cm of the EPR tube with frozen particles using the sucking method.

### The Myoglobin-Azide Reaction: Rate Constant and Freezing Time

For the test reaction, freeze-quench samples were prepared by mixing 4.8 mM myoglobin with 60 mM sodium azide, both in 100 mM sodium phosphate buffer at pH 7.8. The experiments were performed with the 2 ml syringes in 1:1 mixing mode and the flow rate of 2.7 ml s^−1^. For each sample, 150 µl of mixed solution was sprayed into isopentane. Since the samples were planned to be used for multi-frequency experiments, 50 μM MnCl_2_ was added to the azide solution. The six-lines pattern of Mn(II) can be used as internal standard for high-frequency EPR measurements. The reaction was allowed to proceed for different times between 2.6 and 8.8 ms before being quenched. The time was calculated from the ram velocity, the length of the aging hose and the mixer volume.

**Fig. 3:**
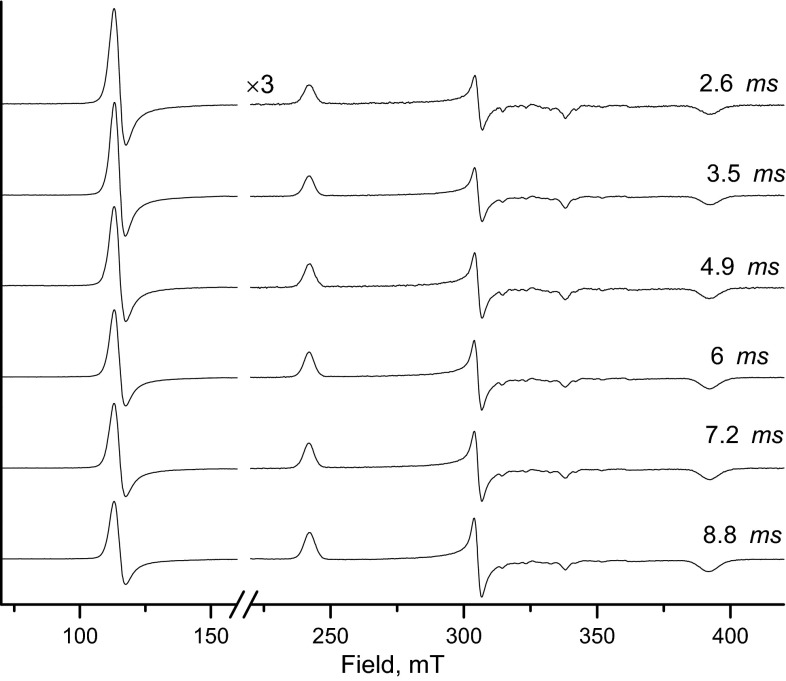
X-band EPR spectra for different reaction times of binding azide to myoglobin. The small signals around 320 mT originate from Mn^2+^, for explanation see the text. EPR measurements: microwave frequency 9.48 GHz, temperature 20 K, microwave power 0.16 mW, modulation amplitude 0.5 mT and time constant of 40 ms

The EPR spectra of the freeze-quench samples are shown in Fig. 3. The decrease (increase) of the HS (LS) signal with time reflects the progress of the binding reaction.

In the presence of a tenfold excess of azide, the binding reaction exhibits pseudo-first order kinetics

$${\text{Ln}} = \frac{{[HS]_{t} }}{{[HS]_{0} }} = - k_{\text{app}} \times t$$

$$k_{\text{app}} = k \times [{\text{N}}_{3}^{ - } ]$$

where *k* is the rate constant and [HS] and [N_3_^−^] are the concentration of HS myoglobin and azide, respectively.

The magnitude of the HS EPR signal depends on the packing density as well as on the amount of the RFQ particles packed in the EPR tube. These vary from sample to sample, which means that the kinetic analysis of the EPR data requires normalization. We consider the ratio of HS to LS signals, which is independent of the density and the sample volume. According to Pievo et al. [29].

$$\frac{{[{\text{HS]}}_{t} }}{{[{\text{HS}}]_{0} }} = \frac{{R_{t} }}{{R_{t} + \lambda }}{ \equiv }Y(t)$$

where

$$R_{t} = \frac{{\left( {I_{\text{HS}} } \right)_{t} }}{{\left( {I_{\text{LS}} } \right)_{t} }}$$

and

$$\lambda = \frac{{\left( {I_{\text{HS}} } \right)_{0} }}{{\left( {I_{\text{LS}} } \right)_{\infty } }}$$

Here (*I*_HS_)_*t*_ and (*I*_LS_)_*t*_ refer to the intensities of the corresponding EPR signals at time *t.*

To properly represent the progress of the reaction, the spectra in Fig. 3 concern the experimental spectra scaled by *Y*(*t*).

**Fig. 4:**
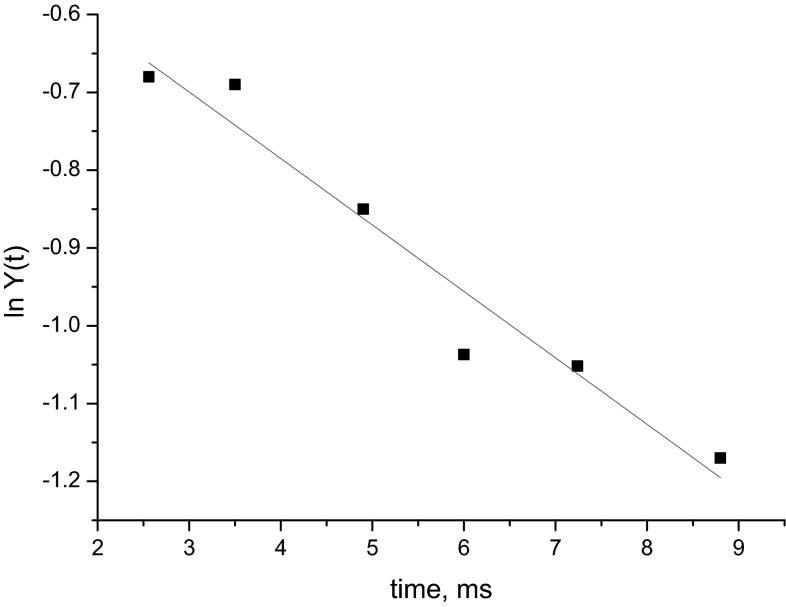
Semilogarithmic plot of *Y*(*t*) as a function of the reaction time. The *line* corresponds to a linear fit of the experimental data

Figure 4 shows the values of ln *Y*(*t*) determined from the EPR intensity of the HS (centered at 115 mT) and LS (at 240 mT) signals as a function of the calculated reaction time. A rate constant *k* = 2.8 ± 0.3 × 10^3^ M^−1^ s^−1^ is derived from the linear fit. Extrapolation results in an intercept of zero for *t* = −5.2 ± 0.7 ms, which corresponds to the freezing time.

## Discussion

The new method to prepare RFQ samples has the advantage of a more quantitative collection of frozen particles in an EPR tube. We find an average collection efficiency of 0.82, which means that only 18 % of the mixed sample does not end up in the EPR tube. In most cases in the literature, no details regarding the collection efficiency are given. In our hands, the amount of reactants required for each RFQ sample is reduced approximately by a factor of three compared to the conventional packing method. This reduction in sample amount becomes more significant considering that 6–10 RFQ samples are needed for a kinetic study. The improved collection efficiency means that less amount of reactants are needed, which is particularly relevant for precious material like proteins of which often limited quantities are available.

The packing factor is not uniquely defined in the literature. In some cases the packing factor is taken as the EPR intensity of a RFQ sample relative to that of the normally frozen sample, in other cases as the ratio between the volume of a RFQ sample in the melted and the frozen state. Anyway, packing factors between 0.4 and 0.7 have been reported [3, 27]. We obtained packing factors of 0.68–0.74 for the sucking method. Moreover, packing by sucking was found more reproducible, which allows a reliable calculation of the amount of the reactants required to achieve an adequate sample in the EPR tube. In the conventional method, the packing factor typically varies by 20 % [3, 27], as compared to 6 % for the sucking method.

In the previously proposed pressure/filtration method [28], use is made of a filter in the EPR tube as used here. Instead of reduced pressure, overpressure is applied and the particle suspension is pushed into the EPR tube. Packing factors 0.45–0.50 have been reported, smaller than for sucking. Moreover, the pressure/filtration method has other drawbacks. It is difficult to estimate the time needed to apply the gas pressure to push the frozen particles into the EPR tube. If not enough isopentane is left in the funnel, the room temperature gas may warm up the sample. Furthermore, the sample tube may detach from the funnel because of a relatively high gas pressure (40–50 psi) and the filter can float while inserting the EPR tube into the isopentane bath.

In contrast to the conventional way of packing, the size of the frozen particles, which depends on the flow rate, is not critical to the collection efficiency and packing time for the new method. The conventional method limits the practical flow rate to less than 1 ml s^−1^ [28], otherwise it is difficult, if possible at all, to accumulate enough frozen particles in the EPR tube. The sucking method permits the use of higher flow rates, as we have shown for the flow rate of 2.7 ml s^−1^. The larger particles formed at lower flow rates, prevent a tight packing. In addition, the effect of electrostatic charge, which is inversely related to the particle size, does not interfere with the collection efficiency using the sucking method. In contrast, the electrostatic charge of smaller particles, which induces repulsion between frozen particles, causes challenges in the collection of sample using conventional packing.

The packing time using the sucking method has dropped to less than 5 min, much shorter than the reported time of about 15 min for the conventional method [28]. The new way to pack the RFQ sample into the EPR tube is convenient to use.

We have obtained the rate constant of 2.8 × 10^3^ M^−1^ s^−1^ for the binding reaction of azide to myoglobin at pH 7.8, which is in agreement with the reported values of 2.5 to 3.0 × 10^3^ M^−1^ s^−1^ under similar experimental conditions [27, 30]. The freezing time of 5.2 ms is obtained with our setup, which is within the range of 5–10 ms reported for similar setups [3, 29, 32]. These agreements demonstrate that the sucking method to pack freeze-quench particles provides adequate samples for kinetic studies by EPR.

In summary, the preparation of rapid freeze-quench samples for X-band EPR benefits substantially from an improved way of collecting and packing of the particles sprayed into isopentane. Sucking of the cold particle suspension into the EPR tube provides superior samples. As compared to the conventional approach, the sucking method requires less material and permits the use of higher flow rates. The sucking method can simply be coupled with a microfluidic RFQ device, for which the dead volume is less compared to that of a standard device. A wider application of the RFQ technique in combination with various forms of spectroscopy becomes feasible. In particular, the sucking procedure can easily be adapted to the preparation of samples for high-frequency EPR, which is presently going on in our laboratory. At microwave frequencies of 95 GHz and higher, EPR tubes can have sub-millimeter diameters, which are difficult to match with standard packing methods.
